# Quantifying Nearshore Sea Turtle Densities: Applications of Unmanned Aerial Systems for Population Assessments

**DOI:** 10.1038/s41598-017-17719-x

**Published:** 2017-12-18

**Authors:** Seth T. Sykora-Bodie, Vanessa Bezy, David W. Johnston, Everette Newton, Kenneth J. Lohmann

**Affiliations:** 1 Duke University Marine Laboratory, Nicholas School of the Environment, 135 Duke Marine Lab Road, Beaufort, North Carolina 28516 USA; 2 Department of Biology, University of North Carolina at Chapel Hill, Chapel Hill, North Carolina 27599 USA

## Abstract

Although sea turtles face significant pressure from human activities, some populations are recovering due to conservation programs, bans on the trade of turtle products, and reductions in bycatch. While these trends are encouraging, the status of many populations remains unknown and scientific monitoring is needed to inform conservation and management decisions. To address these gaps, this study presents methods for using unmanned aerial systems (UAS) to conduct population assessments. Using a fixed-wing UAS and a modified strip-transect method, we conducted aerial surveys along a three-kilometer track line at Ostional, Costa Rica during a mass-nesting event of olive ridley turtles (*Lepidochelys olivacea*). We visually assessed images collected during six transects for sea turtle presence, resulting in 682 certain detections. A cumulative total of 1091 certain and probable turtles were detected in the collected imagery. Using these data, we calculate estimates of sea turtle density (km^−2^) in nearshore waters. After adjusting for both availability and perception biases, we developed a low-end estimate of 1299 ± 458 and a high-end estimate of 2086 ± 803 turtles km^−2^. This pilot study illustrates how UAS can be used to conduct robust, safe, and cost-effective population assessments of sea turtle populations in coastal marine ecosystems.

## Introduction

Sea turtles represent some of the most ancient and enduring megafauna species on the planet. Despite successfully surviving shifting environmental conditions through time^1^, global sea turtle populations have been depleted by human activities such as hunting, egg poaching, habitat destruction, and commercial fisheries^2–4^. Encouragingly, some populations have weathered these pressures and have stabilized or begun to recover^5–7^. This recovery can be attributed, in part, to improved fishery management techniques, the development of conservation projects, and the prohibition of trade via the Convention on International Trade in Endangered Species of Wild Fauna and Flora^8^. While global conservation efforts are beginning to pay dividends in some locations^5–7,9^, many populations of sea turtles face a longer path to recovery^10–12^. Thus, continued monitoring is necessary to inform population assessments and subsequent management decisions.

Baseline data on sea turtle density and distribution are needed to ensure that fishery regulations are appropriately implemented and conservation efforts are effectively designed so that species can fulfill their ecological roles. Historically, sea turtle population data (e.g., numbers of hatchlings and net recruitment) have been obtained by assessing nesting beach productivity, carrying out in-water captures, and conducting aerial surveys with occupied aircraft^13^. Such methods, however, are often time-consuming, labor-intensive, and costly. As an alternative or complementary technique, researchers have increasingly employed fixed-wing and vertical takeoff and landing unmanned aerial systems (UAS) as data collection platforms for monitoring populations^14^. These new approaches can be safer, more cost-effective, reduce the likelihood of disturbance and behavioral impacts, and have the potential to significantly improve the accuracy of population surveys^15^. Although the information collected can be in the form of biological samples or photogrammetric measurements used for individual health assessments (e.g., growth and body condition), we restrict our discussion here to the high-resolution imagery commonly used in population censuses^16–18^.

Several recent studies demonstrate the viability and effectiveness of UAS for studying marine wildlife at sea with a variety of approaches. For example, line transect surveys in Shark Bay, Australia conducted using a fixed-wing ScanEagle UAS identified large marine megafauna and accurately classified 95% of possible dugongs as “certain” sightings^19^. Similarly, a TD100E UAS equipped with GoPros was successfully used to collect bowhead whale images for use in a photo identification mark-recapture study; no detectable responses to UAS presence were observed^20^. In another study, Bevan *et al*. effectively used a DJI Phantom equipped with a GoPro to collect live video feed and observe the courtship and mating behavior of Kemp’s ridley turtles^21,22^. Additionally, objects placed throughout the study area at various depths beneath the water surface were successfully identified, thus increasing confidence in data quality^21^. These studies demonstrate the feasibility of collecting reliable population and behavioral data from marine animals with UAS technology.

The present study explores methods for conducting population surveys and estimating sea turtle densities in nearshore habitats using a small, fixed-wing UAS. Specifically, we present results from a pilot study conducted at a well-known olive ridley turtle (*Lepidochelys olivacea*) nesting beach—Ostional Beach, at the Ostional National Wildlife Refuge in Costa Rica—which hosts some of the largest mass-nesting events of sea turtles in the world^23^. Despite Ostional’s status as a nesting hotspot, studies have not been conducted on the nearshore distribution of sea turtles at this site since 1970 and little is known about the density of sea turtles in nearshore waters^24^. The relatively predictable, large aggregations of sea turtles in nearshore waters make this a promising model system in which to test the use of UAS for aerial population assessments^25^.

## Methods

### Ethics Statement

This study was conducted under permits from the Costa Rican government (Permit # ACT-OR-DR-048-15) and approval of the University of North Carolina at Chapel Hill IACUC (15-220.0-A). The study was designed to avoid disturbances to sea turtles and we recorded no noticeable changes in behavior in response to UAS during aerial surveys.

### Data Availability

All original imagery used in completing this analysis is available from the authors upon request. More information available at the Duke University Marine Robotics and Remote Sensing Lab webpage: http://superpod.ml.duke.edu/uas/.

### Study Area

**Figure 1:**
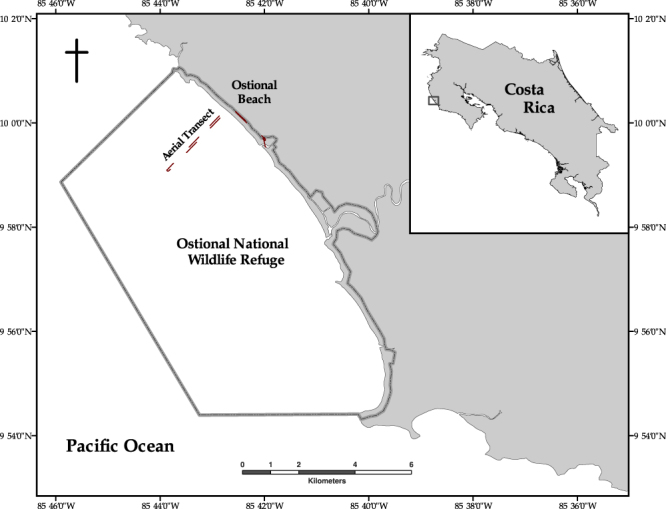
The Ostional National Wildlife Refuge in Costa Rica with the take-off point, transit path, and 3 km aerial transect shown in red. Map created in ArcGIS Desktop: Release 10.4.1, Environmental Systems Research Institute.

The Ostional National Wildlife Refuge is an 86 km^2^ coastal wildlife refuge located on the northern Pacific peninsula of Costa Rica (Fig. 1). The refuge extends 200 meters inland from the high tide line and approximately 5.5 km offshore. Mass-nesting events occur at Ostional Beach almost every month of the year and peak nesting season coincides with the rainy season (May–November).

### Aircraft and Sensors

**Figure 2:**
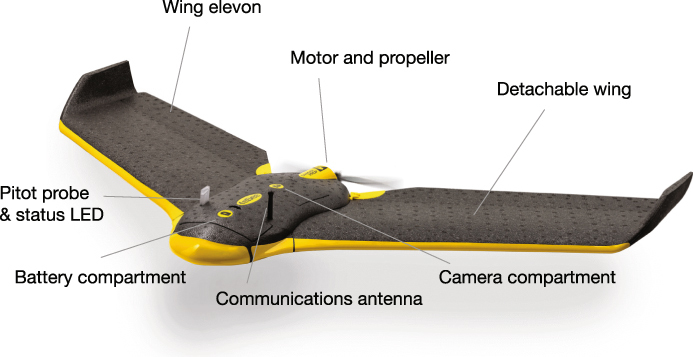
A schematic of the eBee small unmanned aerial system with labels indicating sensors and major components. Figure created by David Johnston.

The eBee (senseFly SA) is a light-weight foam, modular, fixed-wing airframe powered by a single electric motor. It can collect data using a combination of visible, near infrared and red-edge imaging systems (Fig. 2). With a wing-span of 96 cm and weight of 0.7 kg, the eBee is highly portable, fitting into a small case and making it well-suited for research in remote locations. The single engine and propeller are rear-mounted to ensure the safety of both the UAS and those operating it. Under normal operating conditions, the eBee will fly for thirty minutes and can survey 1 km^2^ at 2–4 cm pixel^−1^ ground resolution and 3–5 cm pixel^−1^ vertical resolution. The aerodynamic profile allows the UAS to cruise at speeds of 36–57 kmh^−1^ and operate in winds of up to 45 kmh^−1^.

Detailed mission planning for each flight was conducted prior to deployment using senseFly’s eMotion 2 software. This software employed wind speed and direction measurements to improve flight performance and flight plans were updated with current atmospheric conditions just prior to launch. After takeoff, the aircraft followed a pre-programmed, 3-dimensional flight path utilizing the internal GPS sensor, a barometer, and pitot tube. The operator used data link communications to monitor the aircraft in flight and to provide any appropriate flight profile corrections. Failsafe logic was programmed within the autopilot to return the UAS to the landing zone if it experienced anomalies in flight performance or extreme wind conditions. The aircraft was launched by hand and recovered after a linear landing within 15 m of a predetermined location. Because the magnetic sand at Ostional Beach contaminated the metallic components of the aircraft and sensors, flights were launched and recovered from nearby sites (i.e., a field adjacent to the beach; Fig. 1).

Although the eBee is a proven terrestrial mapping platform, in this study we specifically deployed the eBee to survey transects over a marine environment. The eBee was equipped with a Canon PowerShot S110 near-infrared (NIR) camera to capture aerial photographs. Previous comparisons between NIR and traditional RGB imagery revealed that NIR imagery provided excellent contrast for visually detecting sea turtles in surface waters.

### Survey Method

The team used a senseFly eBee aircraft to conduct surveys at Ostional Beach, Costa Rica on August 6, 7, 8, and 9, 2015, following the start of an olive ridley turtle mass-nesting event during the night of August 5, 2015. A modified strip-transect method was employed to conduct aerial surveys along a three-kilometer trackline over nearshore waters. The transect initiated near the area of the beach where nesting was most concentrated (9.996°N, 85.698°W) and stretched 3 km perpendicular to the shoreline (Fig. 1). To reduce the potential for double-counting turtles along the flight path, we used imagery overlap of 35–45% latitudinally (frontlap), and assessed even and odd images as two separate samples of each transect (hereafter referred to as “sample 1” and “sample 2”), negating the possibility of double-counting individual sea turtles located near the edges of images. Outbound and inbound flights were conducted with minimal (10%) longitudinal (sidelap) overlap to provide 2 independent but adjacent surveys for the study area.

**Figure 3:**
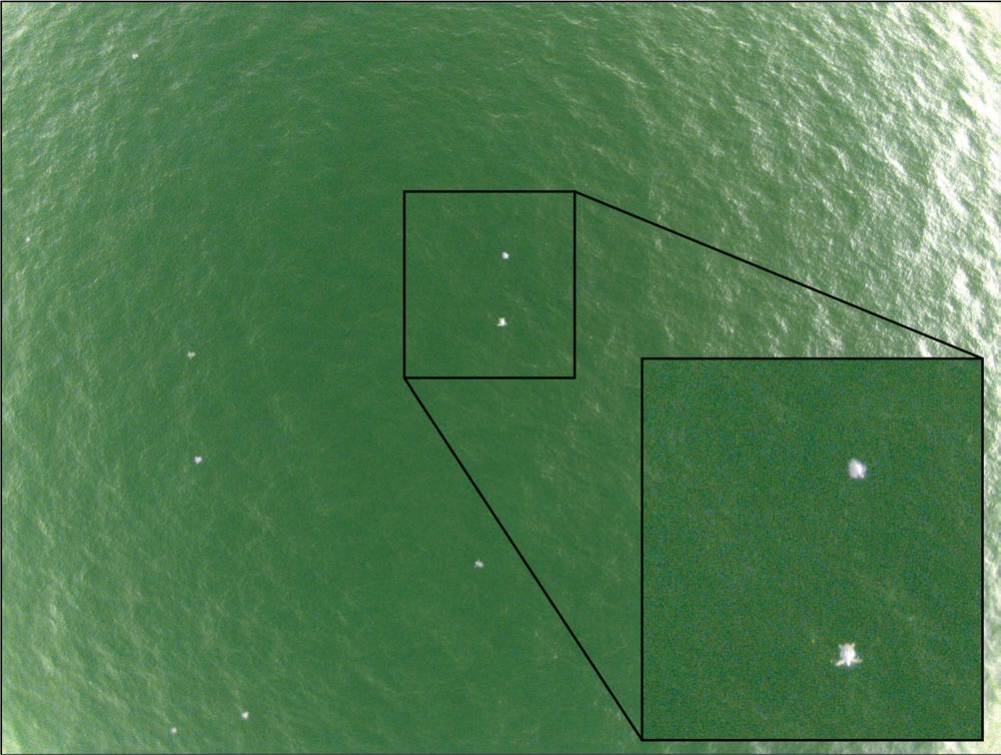
An example of a near-infrared photo of olive ridley sea turtles obtained from the eBee fixed-wing UAS during a survey (90 m altitude, 2.5 cm resolution).

The transects were flown at an altitude of 90 m to maximize the area contained within each image while retaining the resolution (2.5 cm) necessary to identify individual sea turtles for a total survey area of 3.03 km^2^ (after adjusting for glare) (Fig. 1). Each flight path took roughly 18 minutes (depending on weather conditions), during which 78 images were obtained that could be used to estimate density (Fig. 3). We conducted a total of six flights from August 6–9, 2015.

### Data and Image Processing

The number of sea turtles in each photograph was first determined by taking an average of manual counts conducted by three independent reviewers based on pre-set criteria. Reviewers used iTag 0.6 to keep track of identified and counted turtles based on visible appendages (“certain”) or similar size, shape, and coloration as sea turtles (“possible”), resulting in “certain” and “possible” counts for each photograph. Logistical tradeoffs such as flight time and survey distance constrained the resolution of UAS imagery, making it difficult to confidently confirm the species of turtle in our photographs. However, we assumed that all individuals were olive ridley turtles due to their relative size and pigmentation as well as their proximity to the nesting beach during a mass-nesting event. While a few Pacific green (*Chelonia mydas*) and leatherback (*Dermochelys coriacea*) sea turtles nest at Ostional Beach, these are distinct in size and pigmentation patterns, and their peak nesting season (October – March) does not coincide with the timing of our study. Although we found the best conditions to minimize glare were solar zenith angles of 20°–50°, some images required post-processing for glare. We used the threshold function and particle analysis tool in ImageJ to determine the area of the photograph obscured by glare. We then used turtle counts and the adjusted (visible) area of photographs to calculate low (certain) and high (certain and possible) end sea turtle density estimates within the study area.

Although basic density calculations require only a population count and study area, robust aerial and shipboard population assessments require the incorporation of availability and perception biases because many turtles are missed by observers^26,27^. The use of remotely sensed images reduces the likelihood of perception bias to near zero by improving the field of view, as well as by changing the angle of observation from approximately horizontal (for an earthbound human) to perpendicular (for an aerial electronic sensor). Nevertheless, this does not solve the challenge of availability bias due to diving behavior and environmental factors such as sea state and turbidity^26^. While less common in sea turtle population assessments, these adjustments are standard in marine mammal population assessments and improve the accuracy of estimates. We used Laake, Calambokidis, & Osmek’s (1997) availability equation (also used in modified forms by Paxton *et al*.^28^, Johnston *et al*.^29^ and Hodgson *et al*.^30^. This equation corrects for availability biases associated with strip-transect surveys of diving marine animals (Equation 1)^28–31^ by using mean surface time (E_s_), mean dive time (E_d_), and observation window (w(x)) to calculate an availability bias (Av).

1 $$Av=\frac{{E}_{s}}{{E}_{s}+{E}_{d}}+\frac{w(x)}{{E}_{s}+{E}_{d}}$$

A review of the literature on olive ridley dive profiles provided estimates of the time spent under water and the time spent at the surface within a given time period (18 hours). Using the average number of dives/surfaces per day (25) as collected from tags, we were able to calculate the mean dive time (35.5 minutes) and mean surface time (E_s_) of 7.43 minutes. Finally, our observation interval was 7.16 seconds (the average time between UAS images)^32,33^. Using these values, we were then able to calculate an availability bias (Av) to adjust initial density calculations (Equation 2). Also incorporated into this calculation was the reduction in total area (A) associated with glare (G). We did not account for turbidity or wind since the vertical angle of analysis and our selected flight times ensured minimum water surface disturbance that was negligible in our images.

2 $${\rm{D}}=(\frac{1}{Av})\times (\frac{n}{A\,\times \,G})$$

We used JMP and R Studio to conduct two-way ANOVAs to determine whether time, flight, sample, or date had any effect on the adjusted certain density counts. Finally, all density estimations presented herein are expressed as means ± standard deviation (SD).

## Results

To determine nearshore abundances of olive ridley sea turtles, the transect flight was conducted six times during which the eBee covered approximately 36 km of total tracklines. Images taken during these surveys covered a combined study area of 3.51 km^2^. However, the total area was affected by glare during certain parts of each flight. After using ImageJ to calculate and remove the area lost to glare, the final study area was 3.03 km^2^. Computing this reduction in area for each individual photo using ImageJ significantly increases the accuracy of our area term (A x G) as opposed to other methods that rely on observers’ estimates.

The total number of turtles detected by reviewers during strip-transects was highly dependent on the sensitivity of the counting method. Conservative, low-end counts that relied on “certain” identifications resulted in a total count of 682 turtles. By contrast, high-end counts that included both certain and “possible” turtles identified by similarities in shape, size, and coloration resulted in a total count of 1091 turtles. When standardized to area, this provided a low-end count of 227 ± 80, and high-end count of 365 ± 140 sea turtles km^−2^ (Table 1).

**Table 1:** The low-end (“certain” counts) and high-end (including “possible” counts) density of sea turtles km^−2^ adjusted standardized to km^−2^.

	Turtles Detected		Adjusted Estimates	
	Low-end	High-end	Low-end	High-end
Mean Density ± SD (turtles km^−2^)	227 ± 80	365 ± 140	1299 ± 458	2086 ± 803
95% Confidence Level (CI)	50.96	89.26	291.19	510.06
Coefficient of Variation (C_v_)	0.35	0.38	0.35	0.38

**Figure 4:**
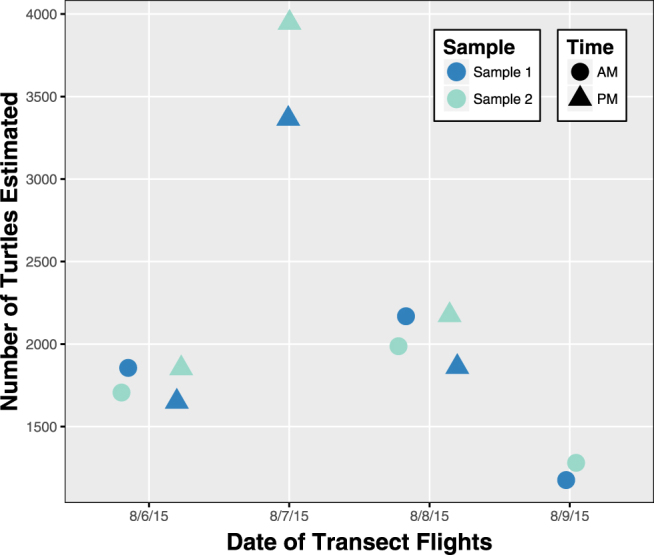
The availability-bias adjusted low-end (‘certain’) estimates of sea turtles during transect flights conducted at Ostional Beach in August 2015. Samples consist of the even or odd images collected during each flight as explained above under the Aircraft and Sensors sub-section of Methods.

Using previously published estimates of time spent in surface waters, we determined (Equation 1) that only 17.5% of the population was ‘available’ during the surveys^33^. As a result, our adjusted estimates indicate a low-end “certain” density of 1299 ± 458 and a high-end “probable” density of 2086 ± 803 sea turtles km^−2^ at Ostional. Additionally, the ANOVA revealed no significant effect of time of day or sample sequence on the number of turtles counted in transects; however, date was a significant predictor of the number of turtles counted during each transect where density spiked during surveys on 8/7/2015 (Fig. 4).

## Discussion

To our knowledge, this study provides the first estimates of nearshore olive ridley turtle densities during a mass-nesting event. More broadly, it illustrates the feasibility of using UAS for conducting large-scale population assessments in remote locations. Given that Ostional hosts some of the largest mass-nesting events of olive ridleys in the world^23,24^, the high density of turtles in nearshore waters at this location may be unique. In-water density data such as these are highly relevant to the establishment and management of marine protected areas such as the area contained within the Ostional National Wildlife Refuge. The approach described in the present study can greatly enhance our knowledge of the density of sea turtles in nearshore waters, improve population assessments, and ultimately benefit sea turtle conservation at larger scales.

### Population Assessment

To ensure reliable population estimates, aerial surveys must incorporate a robust understanding of the specific population’s dive times, foraging habits, and nesting behavior, given that these may vary by species, within species, or based on environmental characteristics (e.g., water depth). For example, loggerheads spend a higher proportion of their time at the surface than olive ridleys^33^ and diving behavior of these species can be dependent on ecosystem type and behavioral state (i.e., as turtles forage, rest, or breed)^34^. Furthermore, mean dive time and surface interval can vary between males and females^35^.

We found very little detailed data on olive ridley turtle diving behavior in relation to sex^35^, water depth^36^ or behavioral/reproductive state. While several studies have investigated olive ridley dive behavior, they do not always report sufficiently fine-scale data needed to calculate availability biases used in our study (Equation 1)^32,37,38^. For the present study, estimates of surface and dive times were derived from a single peer-reviewed study of olive ridley diving behavior in the open sea^33^. While these data may accurately represent at-sea behavior of olive ridleys, they may not completely capture variability in diving behavior of this species shortly before or after nesting^35,36^. As such, future studies should assess dive behavior in nearshore waters of nesting areas to refine population estimates generated through UAS surveys. To develop scalable population estimates, future studies should also tailor their sampling methods accordingly, e.g., fly longer transect patterns distributed across larger geographical areas and longer periods of time. Additionally, UAS surveys should be paired with in-water surveys to determine male to female sex ratios. Addressing these lines of inquiry will improve the accuracy of density calculations and population assessments^39^.

### Application of UAS Technology

The need for safe, cost-effective, and accurate survey methods has made UAS an increasingly common tool for conducting population assessments in support of conservation interventions, fisheries management, and other types of regulatory decisions. In addition to providing a quick and easy method for collecting significant amounts of data at a comparatively lower cost, UAS can also approach closer than traditional aerial and shipboard surveys, providing higher resolution data that is more reliable due to the reduction in perception bias. Although this study shows that UAS surveys can stand alone as a methodological approach, they can also be used in combination with other traditional survey methods^14^. By combining data from multiple methods, researchers will be able to identify standard corrections for converting between datasets collected using different survey methods.

Our experience and others’ (see reviews: Durban *et al*., 2015^16^; Rees *et al.* In Press^14^; Smith *et al*. 2016^15^) indicate that impacts from UAS are minimal in comparison to those caused by other data collection methods (e.g., manned aircraft, boat-based surveys, in-water captures). At no point during our fieldwork did we observe any indication of behavioral disturbances of sea turtles from exposure to the UAS. However, it is possible that their operation may have unobservable adverse impacts on sea turtles’ physiology (e.g., elevated heart rates)^40^. We encourage researchers to minimize the opportunity for wildlife disturbance by safely and responsibly operating UAS. We also suggest that researchers follow Hodgson and Koh’s recommendation to “adopt the precautionary principle in lieu of evidence” when utilizing UAS for wildlife monitoring and research^41^.
