# Author Correction: Quantifying Nearshore Sea Turtle Densities: Applications of Unmanned Aerial Systems for Population Assessments

**DOI:** 10.1038/s41598-018-24799-w

**Published:** 2018-04-19

**Authors:** Seth T. Sykora-Bodie, Vanessa Bezy, David W. Johnston, Everette Newton, Kenneth J. Lohmann

**Affiliations:** 1 Duke University Marine Laboratory, Nicholas School of the Environment, 135 Duke Marine Lab Road, Beaufort, North Carolina 28516 USA; 2 Department of Biology, University of North Carolina at Chapel Hill, Chapel Hill, North Carolina 27599 USA

Correction to: Scientific Reports 10.1038/s41598-017-17719-x, published online 18 December 2017

The original version of this Article contained errors. In the Abstract,

“After adjusting for both availability and perception biases, we developed a low-end estimate of 1299 ± 458 and a high-end estimate of 2086 ± 803 turtles per km^−2^.”

now reads:

“After adjusting for both availability and perception biases, we developed a low-end estimate of 1299 ± 458 and a high-end estimate of 2086 ± 803 turtles km^−2^.”

In the Results section,

“When standardized to area, this provided a low-end count of 227 ± 80, and high-end count of 365 ± 140 sea turtles per km^−2^ (Table 1).”

now reads:

“When standardized to area, this provided a low-end count of 227 ± 80, and high-end count of 365 ± 140 sea turtles km^−2^ (Table 1).”

In Equation 2,

$${\rm{D}}=Av+(\frac{n}{A\,x\,G})$$

now reads:

$${\rm{D}}=(\frac{1}{Av})\times (\frac{n}{A\times G})$$

In the Reference list originally published, References 6 and 7 were duplicated as References 11 and 9 respectively, the author list in Reference 27 was incomplete, and volume and page numbers were missing from many of the references.

These errors have now been corrected in the PDF and HTML versions of this Article.

